# Free and Bound Phenolic Compounds Present in Orange Juice By-Product Powder and Their Contribution to Antioxidant Activity

**DOI:** 10.3390/antiox11091748

**Published:** 2022-09-03

**Authors:** María del Mar Camacho, Mónica Zago, Eva García-Martínez, Nuria Martínez-Navarrete

**Affiliations:** Food Investigation and Innovation Group, Food Technology Department, Universitat Politècnica de València, Camino de Vera s/n, 46022 Valencia, Spain

**Keywords:** free phenols, bound phenols, antioxidant activity, orange juice by-product

## Abstract

Interest in fruit/vegetable consumption is not always linked to a particular diet but rather derives from their high antioxidant activity (AOA), with potential health benefits provided, in part, by polyphenols. Although phenols can be found in free form (FP) or bound to plant tissues (BP), the experimental methodology most frequently used for the quantification of total phenols (TP) is based on the extraction of a portion of FP, which may justify the lack of correlation often found between them and AOA. In this context, four successive extractions were performed to obtain FP and BP of powdered orange juice by-product, and their contribution to the AOA was studied. The first extract (MeOH, 30 °C) can be assumed to be one of the conventional methods for the quantification of TP. Re-extraction with MeOH (60 °C) afforded the FP. Two subsequent basic and acidic extractions yielded the BP. Although the FPs were the most abundant, the AOA (DPPH method) of the last fraction of BP was of the same order found in the first fraction of FP. This highlights the interest in extracting the BP from the by-product of orange juice if its antioxidant capacity is to be exploited.

## 1. Introduction

Interest in diets rich in fruits and vegetables has increased in recent years due to their antioxidant properties and numerous benefits for human health. The choice of fruit consumption is no longer based on personal taste, but on trying to achieve the healthiest lifestyle possible. This has led many companies to develop products that include natural antioxidants in their formulations [1]. Part of this antioxidant capacity is provided by polyphenols, secondary metabolites specific to the plant kingdom. These polyphenolic structures include a wide variety of compounds that differ from each other by the position and number of hydroxyl groups and by the variety of functional groups they present (methyls, sugars, organic acids) [2]. Among them, the most common are phenolic acids and flavonoids [3]. Flavonoids are exogenous compounds that must be acquired through the diet and are considered non-enzymatic antioxidants and are specifically classified as primary antioxidants [4]. They include flavonols, flavones, and flavanones as the main subgroups.

Phenols, among other functions, are structural components of cell walls, which provide coloring and flavoring properties to vegetables and act as protectors of wood, cork, seeds, and other plant structures [5]. In addition, there are scientific studies proving that polyphenols have several benefits in the human organism. Their antioxidant, anti-inflammatory, antithrombotic, and antimicrobial capacities have been shown not only to decrease the risk of chronic diseases such as cardiovascular disease or cancer, but also to delay the spread of disease and facilitate the healing process [1,3,6,7].

Different fruits are widely recognized for their high phenolic content, such as blackberries, blueberries, and red grapes, with reported values of 447, 311, and 170 mg gallic acid (GA)/100 g, respectively [8]. In the case of citrus fruits, TP was reported in the range of 310.18 to 575.06 mg GA/100 g for the corresponding peel and is higher than those of edible fruit tissues (109.16 to 118.94 mg GA/100 g) [9,10]. In addition, the pulp fraction primarily contains flavonoids in the form of glycosides, while citrus peel is also abundant in the less polar flavanone as well as flavone aglycones and polymethoxyflavones, these being the most hydrophobic among flavonoids and present in oil glands of citrus peel [11].

**Figure 1:**
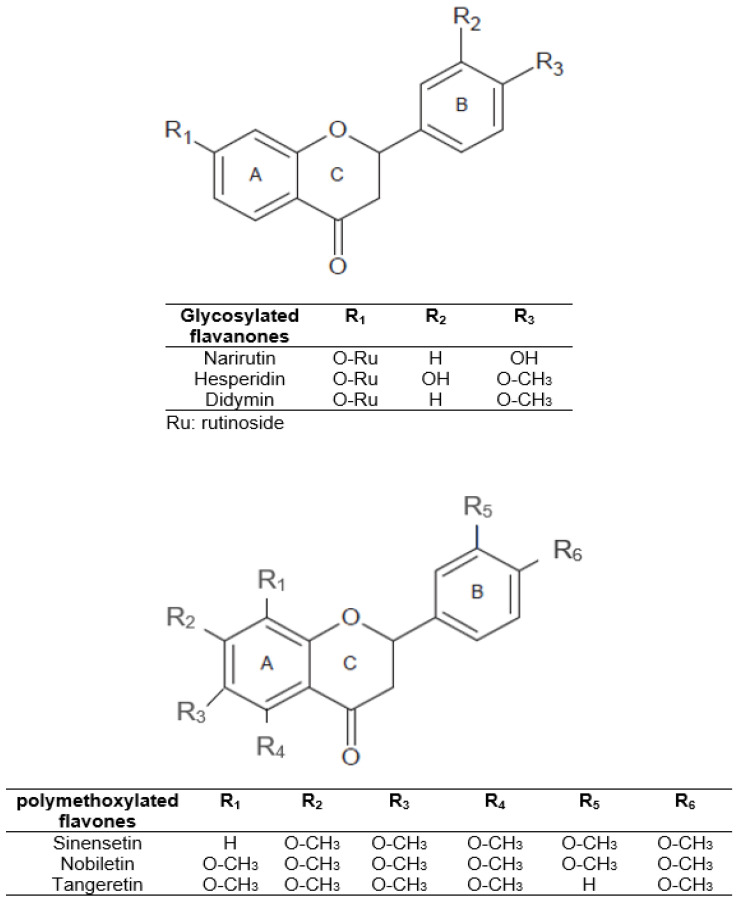
Structure of the main citrus peel flavonoids.

In view of the above, we can understand the interest in obtaining phenolic compounds from by-products of plant origin, thereby contributing to the circular economy of the agri-food sector. Citrus peel can be a source of economic and environmental problems due to fermentation and microorganism spoilage processes. However, citrus peel could be a valuable by-product of citrus industries that can be used in food, pharmaceutical, or cosmetic industries. It is a promising supply of natural flavonoids, dietary fiber, and minerals. Therefore, citrus peel could be employed in food products as a functional ingredient for potential health properties and/or as a substitute for chemical preservatives. In citrus fruits, specifically in orange peel, the main flavonoids are glycosylated flavanones such as narirutin, hesperidin, and didymin [12]. In addition, polymethoxylated flavones such as sinensetin, nobiletin, and tangeretin are present in smaller amounts [4]. The structures of these major compounds are shown in Figure 1.

However, it should be noted that phenols can be found in free form or bound to plant tissues. The latter are associated with polysaccharides (dietary fiber), proteins, or are simply bound to each other, forming high-molecular-weight compounds, which makes their extraction difficult [13,14]. For this reason, it is important to develop methods that allow the extraction of all phenolic compounds. This will make it possible to identify, for each food, the proportion of free and bound phenolics. To extract free polyphenols, there is a consensus in most methodologies. A single extraction is performed using an aqueous organic solvent, such as MeOH, either in pure form or with mixtures of solvents (ethanol, propanol, acetone, water) [13,15]. Some recent examples, although many others could be cited, are the studies of Grande-Tovar et al. [16], who left fruit to macerate with a hydromethanolic solvent and ethanol in different stages; Herrera et al. [17], where antioxidant activity was determined directly from fruit nectar without any prior extraction; and Solomaku et al. [18], who carried out a solvent extraction of solids without the use of heat and using ethanol as the solvent. In all these examples, generally, a single extraction step is performed in which no heat is used. It seems that the increase in temperature produces a higher solubility of the compounds and increases their rate of transfer, since the viscosity and surface tension decrease, but many of the phenolic compounds can be oxidized [19]. Nevertheless, as pointed out by Valdés-Hernández et al. [20], temperatures in the range of 50–70 °C may be sufficient to aid extraction. On the other hand, in none of the above studies were acidic or basic reagents used to carry out the hydrolysis reactions which facilitate the release of bounded compounds. Condensed tannins, hydrolyzable polyphenols, and high-molecular-weight compounds may be found in the bound fraction of polyphenols and cannot be extracted with solvents, requiring acidic, basic, or enzymatic hydrolysis [14]. The acidic and/or basic hydrolysis of the ester binding of these compounds allows the release of the polyphenols bound to the plant matrix and, on the other hand, allows the depolymerization of high-molecular-weight compounds to compounds of simpler structure. In this case, the degradation of certain polyphenolic structures, such as benzoic and hydroxycinnamic acids, may also take place [19].

The objective of this study was to quantify the free and bound phenolic compounds of orange juice by-product powder and their contribution to the antioxidant capacity. In addition, the major flavonoids present in each phenolic fraction were identified. The obtained results highlight the interest in also extracting the bound phenols from orange juice by-product if its antioxidant capacity is to be exploited.

## 2. Materials and Methods

### 2.1. Obtaining the Orange Juice Powdered By-Product

The orange juice by-product used as a raw material for this study was obtained (Speed Up, Zumex, Valencia, Spain) from the cafeteria of the Facultat de Belles Arts Sant Carles at the Universidad Politécnica de València (Spain) in February 2022. After thoroughly removing the stalks and seeds, each half of the orange juice by-product was cut into 4 portions and mixed with water (100:37.8, w/w) to ensure proper handling during emulsification for 5 min (Robot coupe blixer2, Valencia, Spain) to obtain a puree. For freeze-drying, this mixture was distributed in aluminum plates (25 cm in diameter, thickness 1 cm) and frozen at −45 °C (Liebherr LGT 2325, Baden-Wurtemberg, Germany) for at least 48 h. The subsequent drying conditions were −50 °C in the condenser, with pressure of 0.05 mbar and shelf temperature of 50 °C, for 21 h (Telstar Lyoquest-55, Barcelona, Spain). The freeze-dried cakes were ground in batches of 40 g with the aid of a food processor (Thermomix TM 21, Vorwerk, Spain) for 20 s at speed 5 (3700 rpm). The powder obtained was sieved (200 μm mesh, AMP0.40, CISA, Barcelona, Spain) and the sample with size >200 μm was ground again (Kenwood CH580, Madrid, Spain) until all the samples had a particle size <200 μm. This powder was analyzed in triplicate for water content (Karl Fisher, Mettler Toledo, Compact Coulometric Titrator C10S, Worthington, OH, USA), and stored in airtight ZIP bags and kept refrigerated (4 °C) until phenol extraction and subsequent analysis (Section 2.2 and Section 2.3).

### 2.2. Extraction of Free and Bound Phenols

Extracts for the determination of free and bound phenolic compounds (FP and BP for free and bound phenolics, respectively) were obtained with the method described by Alu’Datt et al. [21]. Briefly, the method is based on four consecutive extractions, the first two to extract FP and the following two to extract BP. For the first extraction, 1 g of sample was extracted with MeOH at 30 °C and filtered (fraction F30). The residue of the filtrate was extracted again with MeOH, this time at 60 °C (fraction F60). The residue of this second extraction underwent basic hydrolysis, and the filtrate, mixed with MeOH, yielded the BB fraction. Finally, an acidic hydrolysis of the previous residue was carried out to obtain the BA fraction after filtering and mixing with MeOH.

### 2.3. Analysis of Phenolic Extracts

We analyzed each of the four phenolic extracts obtained as described in Section 2.2 in triplicate for the total phenol content (TP), major flavonoids, and antioxidant activity (AOA). For the quantification of the phenolic content, the modified Folin–Ciocalteu spectrophotometric method according to Alu’Datt et al. (2017) [21] was used. The measurements were taken using a UV–Vis spectrophotometer (V-1200 VWR, VWR, Radnor, PA, USA) and the phenolic content was expressed as mg GAE/100 g dry basis (db). The flavonoid profile of the extracts was analyzed by UHPLC (Jasco equipment, Cremella, Italy), using a Synergi 4 μm Hydro-RP column (Phenomenex, Valencia, Spain). MeOH and HPLC-grade H_2_O were used as the mobile phase. The flow rate was 1 mL/min and the injection volume was 10 μL. Once the profiles were obtained, identification was performed at a wavelength of 284 nm, and for quantification, the proper patterns were used (TCI Europe N.V., Paris, France). The AOA was determined by the DPPH (1,1-diphenyl-2-pricrylhydrazyl) method [22]. The results were converted to mmol Trolox equivalent (TE)/100 g (db) using a standard curve of Trolox (Sigma-Aldrich, Steinheim am Albuch, Germany) and the same spectrophotometer described above.

### 2.4. Statistical Analysis

To determine the significant differences between the different phenolic extracts, a simple statistical analysis of variance (ANOVA) was performed using Statgraphics Centurion version XVIII software with a confidence level of 95% (*p*-value < 0.05). Data obtained from each extraction were also explored and modeled using principal component analysis (PCA). The same program was used to study the Pearson correlations among the studied compounds and the AOA.

## 3. Results

The water content of the orange juice by-product powder was 3.60 ± 0.12 g H_2_O/100 g per sample.

### 3.1. Total Phenolic Compounds

Table 1 shows the content of total phenols present in the free and bound fractions extracted from the orange juice powder by-product, totaling 669.9 mg GAE/100 g of powdered by-product. The sum of F30 and F60, 539.58 mg GAE/100 g of orange juice by-product powder, is free phenols. The sum of BA and BB, 130.31 mg GAE/100 g of by-product powder, corresponds to bound phenols. Of the four fractions of phenolic compounds extracted, F30 is what we could call “conventional” since it was obtained at 30 °C and using MeOH, similar to the methodology normally described for the analysis of total phenolics in food products. In fact, F30 allowed us to extract the highest TP content.

**Figure 2:**
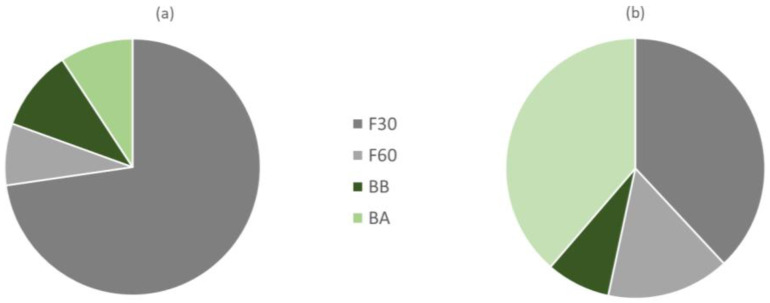
Proportion of total phenols (**a**) and antioxidant activity (**b**) associated with each of the phenol fractions extracted (codes according to Section 2.2).

The results obtained indicate that if we would have kept only the F30 fraction for the characterization of the total phenols of the orange juice by-product powder, we would be losing 27.2% of the TP in the sample, of which 7.8%, 10.2%, and 9.2% would be in fractions F60, BB, and BA, respectively (Figure 2a). On the other hand, the two extractions with MeOH at 30 and 60 °C allow the extraction of all the free phenols, which account for 80.6% of the total, with the remaining fraction represented by bound phenolics that required basic and acidic extraction for 24 h for release. These findings are consistent with Alu’datt et al. [21], who showed that most phenolics are found in the free form in different fruits of the Rutaceae family (85–93% of the total phenolic content).

### 3.2. Main Flavonoids

**Figure 3:**
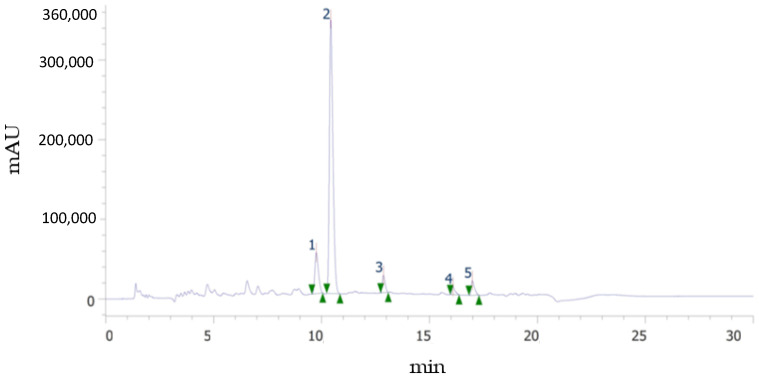
Example of chromatogram showing the flavonoid profile of the sample. (1) Narirutin. (2) Hesperidin. (3) Didymin. (4) Sinensetin. (5) Nobiletin.

As it is well known, spectrophotometric methods are useful for an approximate estimation of total polyphenol and total flavonoid contents, as well as antioxidant capacity, it being necessary to employ more selective and robust methods, such as chromatographic ones, to obtain the individual polyphenolic profile. Figure 3 shows an example of an UHPLC chromatogram at 284 nm, in which the peaks corresponding to the flavonoids identified in this study are observed: narirutin (NAT), hesperidin (HES), didymine (DID), nobiletin (NOB), and sinensetin (SIN). It should be noted that although these flavonoids are considered the major ones, DID, SIN, and NOB were present in quantities much lower than NAT and HES. The glycosylated flavanones (HES, NAT, and DID) and the metoxylated flavones (SIN and NOB) identified in this study eluted in the order reported by other authors [23,24], i.e., more polar NAT, HES, and DID eluted first because their sugar moiety increased their water affinity, while less polar methoxy groups in SIN and NOB lowered their solubility and thus appear later in the chromatogram, at longer retention times. SIN, with five methoxy groups, eluted before NOB, with six groups. Quantification was possible for HES and NAT in all phenolic fractions, mainly in F30, while SIN and NOB were not found in the bound fraction.

**Figure 4:**
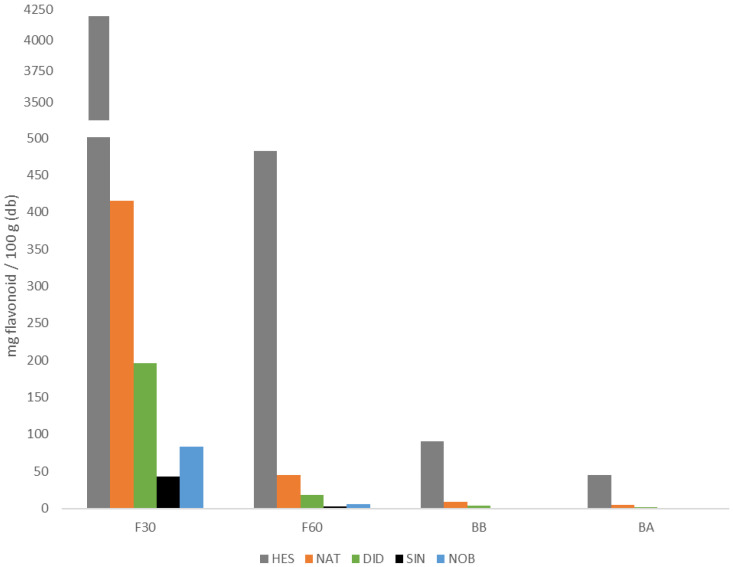
Flavonoid content of narirutin (NAT), hesperidin (HES), didymin (DID), sinensetin (SIN), and nobiletin (NOB) in the different phenolic extracts studied (codes according to Section 2.2).

Figure 4 shows the content of each of the flavonoids identified in each of the four extracts analyzed and Table 1 shows both the sum of all of them for each fraction (Fi) and the sum of all four fractions, the latter being the total amount of flavonoids identified in the powdered orange juice by-product. The main flavonoids found in each phenolic fraction were hesperidin and narirutin. As can be observed, the highest amount of flavonoids was found in F30, followed by the F60 free phenolic fractions. Between 90% and 95% of each of the quantified flavonoids (NAT, HES, DID, SIN, and NOB) were found in the free fractions, being between 86% and 94% in F30. Although the bound flavonoids present in the BA and BB fractions do not contribute much to the total, it is sufficient to take them into account, especially the BB fraction.

### 3.3. Antioxidant Activity

To further evaluate the functionality of each polyphenol fraction, DPPH radical scavenging activity, which is the most representative indicator reflecting the antioxidative activity of a plant extract, was determined [25]. This assay involves an electron transfer reaction, which tests the reducing power of antioxidants [26]. Oxidation reactions (involving the shifting of electrons among electron rich species or molecules) produces free radicals, mostly reactive oxygen species. These species may damage many biomolecules of the cell and additionally might be responsible for degenerative illnesses such as multiple sclerosis and cancer in humans [10].

Table 1 shows the antioxidant activity of all four phenolic extracts obtained in this study. The antioxidant activities of F30 and BA extracts were significantly higher (*p* < 0.05) than F60 and BB extracts. It is convenient to note at this point that the F30 fraction was the one with the highest TF and Fi content and the BA was the one with the lowest TF and Fi content. The distribution of antioxidant activity associated with each of the free and bound fractions of total phenols extracted indicates that the F30 and BB fractions provided 38% and 38.7% of the total antioxidant activity of orange peel, respectively (Figure 2b). Of the rest, 15.2% of the AOA was found in F60 and 8.1% in BB extracts. Zou et al. [27] and Alu’datt et al. [21] found that bound phenolics extracted had higher antioxidant activity than free phenolics extracted from citrus fruits.

### 3.4. Statistical Correlations

**Figure 5:**
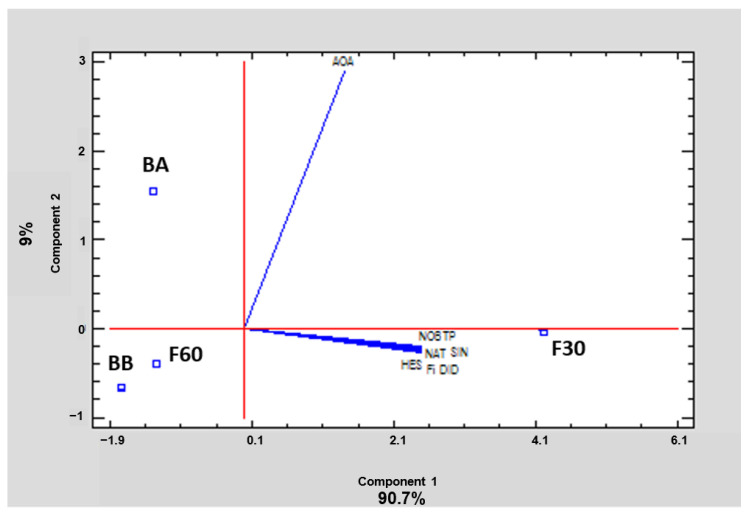
Principal component analysis of total phenols (TP), sum of identified flavonoids (Fi), hesperidin (HES), narirutin (NAT), didymin (DID), nobiletin (NOB), sinensetin (SIN), and antioxidant activity (AOA) of the four polyphenol fractions (codes according to Section 2.2) of orange juice by-product powder.

In order to summarize and easily visualize the results from all polyphenolic fractions, data were subjected to PCA. This tool reduces the dimensionality of the multivariate data to two or three principal components that can be visualized graphically, with minimal loss of information. In this case, two principal components explained 99.7% of the variance. Relationships between samples and variables were investigated. The 2D graph obtained (Figure 5) revealed patterns and differences among polyphenol orange peel extracts. Component 1 explained 90.7% of the variability, and showed a positive correlation with all the variables analyzed. Component 2 explained 9% of the variance, and showed a negative correlation with polyphenols and a positive correlation with AOA. As can be observed, the loading directions for vectors of TP, the identified flavonoids, and the sum of the latter are the same, but different from the loading directions of the AOA vector. This suggests that the behavior of phenolics is similar and they are well correlated, but not with AOA. On the other hand, the proximity of F30 extract to the loading vectors corresponding to TP, Fi, and the amount of each of the identified flavonoids revealed that F30 was the fraction with the highest amount of these compounds, as also can be seen in Table 1. Figure 5 also confirms the important correlation between DPPH antioxidant capacity and BA extract, showing that this bound fraction has the highest AOA.

Table 2 shows the Pearson correlation coefficients (r) between TP, Fi, the amount of each identified flavonoid, and AOA considering the data of all four extracts together. A strong positive significant correlation (*p* < 0.05) between TP, Fi, and the amount of each identified flavonoid was found. Nevertheless, there was no significant correlation (*p* ≥ 0.05) between antioxidant activity and any of the phenolics. Similar results were observed by Alu’datt et al. [21] for different fruits of the Rutaceae family. As has already been noted, the main flavonoids found in each phenolic fraction were the glycosylated flavanones hesperidin and narirutin, but it is known that flavanones do not belong to the best antioxidant family. This could suggest that the chemical structure of each individual antioxidant, and not just their concentration, influences the antioxidant activity. In contrast, a significant correlation (0.9757 ≤ r ≤ 1.0000; *p* < 0.05) was obtained between AOA and the rest of variables when only data from the free polyphenols (F30 and F60 fractions) were considered. This may indicate that there are other unassessed phenolic components in the BA fraction that affect the antioxidant capacity of orange by-product.

## 4. Discussion

The results obtained from the analysis of total phenols of orange peel show the convenience of using more specific extraction methods than those conventionally used to extract not only free phenolic compounds but also those bound to other cellular structures, which represent about 20% of the total phenols and appear to contribute to the antioxidant capacity of the product to a large extent. Although the flavonoid profiles identified in this study in the extracts of the free and bound fractions were similar, the TP content was lower in the latter than in the former, while the AOA was higher in the latter. A higher DPPH radical scavenging capacity of bound phenolic compounds was found by Gulsunoglu et al. [28] in chestnut and apple residues and by Wang et al. [29,30] for seed oil.

The main bound phenolic compounds present in orange peel could be the hydroxycinnamic acids caffeic, p-coumaric, ferulic, and sinapic [31,32]. Ferulic and p-coumaric have been quantified as major phenolic acids and caffeic acid as a minor phenolic acid in peels of citrus fruits (lemons, oranges, and grapefruits), and their levels were significantly larger than those of peeled fruits [33,34,35]. In other studies, ester derivatives of ferulic and sinapic acids (dihydroxycoumarin, dihydroxycoumarin-O-sinapoyl-glucose ester, and feruloyl glucoside ester) were also identified in orange peel [36]. Citrus phenolic acids show strong antioxidant properties through the dehydrogenation of hydroxyl groups and the effect of ortho-substitution on a benzene ring [27]. For orange peel, caffeic acid is the least abundant compound, whereas it has the highest AOA value compared to the rest of phenolic acids [33]. Nevertheless, Bocco et al. [33] also observed that the phenolic acids in orange peel explain only from 2% to 22% of the AOA of the extracts. This means that some other substances are surely responsible for the antioxidant capacity of the peel extracts. The interaction or synergistic effect among the bioactive compounds contained in citrus fruits may also contribute to their antioxidant capacity. In the current literature, the interaction between naringenin and hesperidin of naval orange (*C. sinensis* Osbeck.) provides an example of the synergism among phenolic compounds [37]. In any case, taking into account all the mentioned studies, and also the fact that due to their chemical structure, methoxylated flavones have a very low antioxidant activity [33], it appears convenient to use extraction methods for free and bound phenols in order to determine the total antioxidant capacity of orange peel.

## Figures and Tables

**Table 1:** Total phenolic content (TP, mg GAE/100 g (db), antioxidant activity (AOA, mmol Trolox/100 g (db)) and sum of the identified flavonoids (Fi, mg/100 g (db)) in the 4 phenolic extracts obtained (codes according to Section 2.2). The sample column corresponds to the sum of the 4 extracts.

	F30	F60	BB	BA	Sample
**TP**	505 ± 36 ^(b)^	54 ± 15 ^(a)^	71 ± 26 ^(a)^	64 ± 7 ^(a)^	694
**TFi**	4933 ± 335	550 ± 3	100 ± 37	51 ± 23	5634
**AOA**	1.18 ± 0.02 ^(b)^	0.47 ± 0.13 ^(a)^	0.25 ± 0.07 ^(a)^	1.2 ± 0.3 ^(b)^	3.1

Different letters ^(a,b)^ in each row indicate different homogeneous groups (*p* < 0.05) according to Tukey’s HSD test.

**Table 2:** Pearson correlation coefficients between total phenols (TP), hesperidin (HES), narirutin (NAT), didymin (DID), nobiletin (NOB), sinensetin (SIN), the sum of identified flavonoids (Fi), and antioxidant activity (AOA) analyzed in the four phenolic extracts of powdered orange by-product.

	AOA	Fi	HES	NAT	DID	SIN	NOB
TP	0.5084	0.9903 *	0.9899 *	0.9910 *	0.9924 *	0.9888	0.9945 *
AOA		0.4940	0.4928	0.4982	0.4997	0.5084	0.5050
Fi			1.0000 *	0.9999 *	0.9998 *	0.9988 *	0.9956 *
HES				0.9998 *	0.9997 *	0.9987 *	0.9953 *
NAT					0.9998 *	0.9985 *	0.9962 *
DID						0.9987 *	0.9964 *
SIN							0.9946 *

* Correlation is significant at *p* ≤ 0.05 level.

## Data Availability

All data used to support the findings of this study are included within the article.
